# Emotion Regulation Difficulties and Smoking Behavior among Adults with Chronic Obstructive Pulmonary Diseases

**DOI:** 10.1007/s11126-024-10080-z

**Published:** 2024-07-15

**Authors:** Mary J. Schadegg, Laura J. Dixon, Aaron A. Lee

**Affiliations:** 1 Department of Psychology, University of Mississippi, P.O. Box 1848, University, MS 38677 USA; 2 Department of Psychiatry, NYU Grossman School of Medicine, One Park Ave, New York, NY 10016 USA

**Keywords:** Emotion regulation, Smoking, COPD, Respiratory disease

## Abstract

COPD is one of the leading causes of death in the United States and results in increased healthcare costs and disability. Smoking is the main determinant of COPD development and continued use increases mortality as compared to those who have stopped smoking. Research has indicated that cigarette smoking may play a role in attempts to regulate distressing emotional experiences and thus, may be an important transdiagnostic process underlying continued smoking behavior among adults with COPD. The current study investigated the role of ER difficulties in relation to smoking status and cigarettes smoked per day among adults with COPD. This cross-sectional study included a sample was adults with COPD (*N* = 320). Participants self-reported current smoking status, daily smoking, and the Difficulties in Emotion Regulation Scale. All analyses were adjusted for age, sex, probable depression, probable anxiety, and dyspnea severity. DERS total scores were associated with greater odds of current smoking. With the exception of impulsivity, all other dimensions of emotion regulation were significantly associated with current smoking. Greater difficulties in emotional awareness were associated with greater cigarettes smoked per day. However, neither the DERS total score nor any other dimensions of emotional regulation were significantly associated with cigarettes smoked per day. The present study provides preliminary data linking ER difficulties to smoking behavior among adults with COPD. If corroborated by future research, these findings suggest that ER might be a potential target for smoking cessation programs among adults with COPD.

**Supplementary Information:**

The online version contains supplementary material available at 10.1007/s11126-024-10080-z.

## Introduction

Chronic obstructive pulmonary diseases (COPD) are a class of chronic progressive respiratory conditions characterized by obstructed airflow and breathing difficulties that contribute to diminished capacity for physical activity [1]. Globally, COPD is a leading cause of death [2], and in the United States (U.S.), COPD is a major source of healthcare costs and disability [3]. Cigarette smoking is the primary cause of COPD disease in the U.S., with 15.2% of current cigarette smokers having COPD compared to 7.6% former smokers, and 2.8% of adults who had never smoked [4]. Individuals with COPD who smoke have accelerated declines in pulmonary function and greater odds of early mortality compared to those who stop smoking [5, 6]. Further, smoking causes eight out of ten COPD-related deaths [7]. Yet, alarmingly, 38% of the approximately 16 million U.S. adults diagnosed with COPD are current smokers [4].

Among adults with COPD, those with comorbid mental health diagnoses are more likely to smoke cigarettes [8]. While research has not specifically investigated the effect of negative affect on smoking behaviors among those with COPD, prior studies have found that smoking is frequently used as a strategy to mitigate the experience of negative affect [9], suggesting a direct connection between smoking and negative emotional experiences. A recent meta-analysis found negative affect has been consistently linked with smoking behavior with small pooled effect sizes (*r*s = 0.06–0.20) [10]. Negative affect has also been associated with greater difficulties in smoking cessation– particularly among individuals struggling with a comorbid emotional disorder [11]. Together, these findings suggest that individuals’ ability to modulate negative affect may be an important risk factor for cigarette smoking among adults with COPD.

Emotion regulation (ER) is a transdiagnostic process that includes the ability to adaptively manage emotional responses [12]. Difficulties in ER can occur across several domains, including awareness and understanding of emotions, acceptance of emotions, ability to control impulses and engage in goal-directed behaviors when experiencing negative affect, and accessing effective strategies that can be used to regulate emotions [13]. Emotion dysregulation has been found to be a stable, trait-like factor that contributes to a range of psychopathology [13, 14], chronic health conditions [15], and substance use disorders [16].

Engagement in substance use to regulate or cope with one’s emotions is well-documented [17], with more recent studies demonstrating the connection between emotion dysregulation and smoking behaviors. Compared to non-smokers, smokers report greater difficulties in ER [18], which is associated with greater negative affect [19, 20]. Among current smokers, emotion dysregulation has been associated with smoking behaviors [21] and the use of cigarettes in an attempt to reduce negative affect [19]. One prior study also found that expectations about the effectiveness of cigarette use for regulating emotions mediated the relationship between depressive symptoms and number of cigarettes smoked per day [22], suggesting that individuals who smoke perceive smoking to be an effective means by which to manage distressing emotional experiences. Past research demonstrates that the association between cravings to smoke and intense negative or positive affect were attenuated when controlling for self-efficacy of ER ability [15]. Further, prior work suggests that ER difficulties increase the risk of relapse among adults who recently quit smoking [23], whereas the use of adaptive ER strategies, such as cognitive reappraisal, results in decreased cravings, lower negative affect, greater positive affect, and increased tolerance for distress [24–26]. Taken together the research suggests that ER difficulties might be a viable treatment target for improving smoking-related outcomes.

Cumulatively, extensive research indicates smoking behaviors are associated with the inability to modulate unpleasant emotional experiences; yet research has not yet elucidated the contribution of ER difficulties in smoking behaviors among adults with COPD. To further understand ER as a potential mechanism in smoking behaviors for this high-risk group, the current study investigated the role of ER difficulties in relation to smoking status and cigarettes smoked per day among adults with COPD. Compared to non-smokers with COPD, we predicted current smokers with COPD would evidence significantly greater difficulties with ER. In addition, overall difficulties in regulating emotions were expected to differentiate smoking status, after adjusting for established predictors such as age, sex, anxiety, depression, and dyspnea severity [1, 8, 27]. With regard to cigarettes smoked per day, we hypothesized greater difficulties with ER would be associated with a higher number of cigarettes smoked per day among smokers with COPD. Finally, exploratory analyses were conducted to identify specific domains of ER difficulties that affected smoking behaviors in this sample.

## Methods

### Participants

Participants were recruited from a large web-based panel of U.S. adults with chronic respiratory disease maintained by Qualtrics. All panel members had previously reported a diagnosis of asthma and/or COPD. Qualtrics panel members were invited to participate by email. To be eligible for this study, interested panel members had to indicate that they were: (1) 18 years of age or older and (2) endorse a current diagnosis of chronic bronchitis, emphysema, or COPD.

A total of 911 adults initiated the survey and 838 (92.0%) provided informed consent to participate in the study. Of those who provided informed consent, 145 reported having no respiratory condition, 229 (27.3%) reported having current asthma only, and 217 reported having a COPD and comorbid asthma. The present study used data from the remaining 320 respondents who reported having one or more COPDs, (i.e., emphysema, chronic bronchitis, or “COPD”) without comorbid asthma.

### Compliance with Ethical Standards

The authors declare that they have no conflicts of interest. This research was supported by funding from Askwith Foundation through the University of Michigan Center for Managing Chronic Disease awarded to AL. The funder did not have any role in the study design; data collection, management, analysis, or interpretation; manuscript writing; or submission for publication. AL and LD’s effort on this paper was supported by the University of Mississippi College of Liberal Arts Research Grant for Faculty Research and Creative Achievement.

This study included human research participants. All study methods and procedures were consistent with the Declaration of Helsinki and approved by the University of Michigan Medical School Institutional Review Board. All participants provided informed consent to participate in this study. Data will be shared by the corresponding author upon reasonable request.

### Measures

#### Emotion Regulation Difficulties

The Difficulties with Emotion Regulation Scale (DERS) [13] is comprised of 36 self-report items that measures a person’s perceived emotional awareness, acceptance of emotions, and ability to regulate emotions. Participants rate each item on a 5-point scale, ranging 1 (*almost never*) to 5 (*almost always*). The total, as well as the six subscales, were separately examined in this study to assess: (a) non-acceptance of emotional experiences (non-acceptance), (b) difficulties in goal-directed behaviors (goals), (c) difficulties controlling impulses when experiencing negative affect (impulse), (d) level of emotional awareness (awareness), (e) access to ER strategies (strategy), (f) level of emotional clarity (clarity), and (g) overall difficulties in perceived ER ability (total). Item responses are summed to compute subscales and the total score with higher scores indicating greater ER difficulties. The DERS has demonstrated good internal consistency (α = 0.93), and good construct validity [13]. In the current study the total and six subscales demonstrated good to excellent internal consistency (αs = 0.80 − 0.95).

#### Smoking Behavior

All participants were asked to indicate their current smoking status using a single item from the National Health Interview Survey (i.e., “Do you now smoke cigarettes, every day, some days, or not at all?”) [28]. Response options included “Yes, every day or some days” or “no, not at all”. Participants who reported smoking every day or some days were asked to indicate the average number of cigarettes they smoke per day (“How many cigarettes do you now smoke on average each day?”).

#### Control Variables

All participants reported their sex and age on a demographic measure to be used as control variables in the present study. The Patient Health Questionnaire-2 (PHQ-2) [29] and the Generalized Anxiety Disorder-2 (GAD-2) [30] were used to determine probable depression and anxiety. The PHQ-2 and GAD-2 each consist of two items which assess critical symptoms of depression and generalized anxiety. Participants rate the extent to which they have experienced each symptom of depression or anxiety on a 4-point scale ranging from 0 (*Not at all*) to 3 (*Nearly every day*). Total scores, ranging from 0 to 6, are computed by summing the responses for each item. A cutoff score of 3 has demonstrated good sensitivity and specificity for accurately identify depression [29, 30] or probable anxiety [31]. Accordingly, in the current study, individuals with total scores 3 on the PHQ-2 or GAD-2 were categorized as having probable depression or anxiety. Dyspnea related impairment was measured using the Modified Medical Research Council (mMRC) Dyspnea Scale [32]. Participants indicate their degree of disability caused by breathlessness during activities by choosing from five responses of increasing severity from 0 (*I only get breathless with strenuous exercise*) to 4 (*I am too breathless to leave the house*). Consistent with previous, mMRC Dyspnea Scale scores were used to classify patients as having mild (< 2) vs. moderate or severe (2) disability [33]. Prior studies suggest that mMRC Dyspnea Scale scores 2 predict lower physical activity [34] and greater risk of early mortality [35].

### Data Analytic Plan

All analyses were performed using SPSS version 27. Descriptive statistics were used to characterize the sample. A series of independent samples t-tests were used to compare DERS total and subscale scores among non-smokers and smokers with COPD. Examination of univariate distributions for each DERS total and subscale did not indicate significant skew or outlying data points. Levene’s test demonstrated equality of variance between groups. Cohen’s *d* was computed as a measure of effect size for each comparison to quantify the difference in DERS total and subscale scores among smokers and non-smokers. Next, we used separate two-step logistic regression models to examine the association between the DERS total and subscales scores with odds of smoking. Finally, we used separate two-step hierarchical linear regression models to examine the association between DERS total and each subscale with total number of cigarettes per day among current smokers. For each multivariable model, control variables, including respondents’ age, sex male = 0, female = 1), probable depression (no = 0, yes = 1), probable anxiety (no = 0, yes = 1), and dyspnea related disability (mild = 0 vs. moderate or severe = 1) were entered in step one and DERS subscales were entered in step two. All tests were two-tailed with alpha = 0.05.

## Results

### Sample Characteristics

Table 1 includes the descriptive statistics for the sample. Most of the sample was male, White, and non-Latinx with an average age of approximately 60 years. Most of the sample had at least some college education and reported an annual income between $15,000 and $55,000. A small portion of the sample screened positive for probable depression (7.2%) or probable anxiety (4.7%). The modal MRC Dyspnea scale score was in the mild range (“I get short of breath when hurrying on the level or up a slight hill”); yet, 45.0% indicated moderate or severe disability. Lastly, approximately half of the sample (*n* = 152) were current smokers who averaged 16 cigarettes per day, which is higher than the prevalence of smoking (38%) among adults with COPD reported by one prior study [36].

**Table 1:** Descriptive statistics of the sample (*N* = 320)

Variable	% or Mean (SD)
Age	61.17 (13.57)
Male	61.9%
Race	
White	88.4%
Black	7.8%
Other	3.8%
Ethnicity (Non-Latino)	92.5%
Education	
< High school	3.4%
High school	25.6%
≥ Some college	70.9%
Income	
< 15,000	14.7%
15,000 to 30,000	30.6%
30,000 to 55,000	29.4%
≥ 55,000	25.3%
MRC Dyspnea Scale	
0	15.0%
1	40.0%
2	30.0%
3	14.1%
4	0.9%
Current smoker	47.2%
Cigarettes/day	15.6 (10.6)
DERS total (*n* = 309)	74.1 (23.5)
Nonacceptance	12.6 (5.8)
Goals	12.0 (4.6)
Impulse	10.6 (4.8)
Awareness	14.3 (4.7)
Strategies	15.4 (6.7)
Clarity	9.1 (3.6)

*SD* = Standard Deviation

### Emotion Regulation Difficulties and Smoking Status

Table 2 depicts the results of *t*-tests comparing DERS total and subscales scores for smokers and non-smokers. As hypothesized, smokers with COPD had significantly higher DERS total scores compared to non-smokers with COPD. Additionally, all subscales for the DERS had significantly higher means for smokers as compared to non-smokers. The difference in DERS scores between smokers and non-smokers were characterized by medium effect sizes (Cohen’s *d* = 0.39–0.67).

**Table 2:** Means, standard deviations, and results of t-tests comparing DERS total and subscale scores among smokers and non-smokers with COPD (*n* = 309)

DERS	Non-Smokers *n* = 160		Smokers *n* = 149				
	*M*	*SD*	*M*	*SD*	*t*	*p*	Cohen’s *d*
Total	66.84	19.32	81.96	25.17	-5.89	<.001	0.67
Non-acceptance	11.29	5.19	14.07	6.16	-4.29	<.001	0.49
Goals	11.13	4.55	13.03	4.45	-3.70	<.001	0.42
Impulse	9.50	3.77	11.88	5.37	-4.48	<.001	0.51
Awareness	13.42	4.41	15.22	4.90	-3.39	.001	0.39
Strategies	13.45	5.36	17.46	7.36	-5.44	<.001	0.62
Clarity	8.06	2.81	10.30	4.03	-5.63	<.001	0.64

*Notes.* DERS = Difficulties in Emotion Regulation Scale, *M* = Mean, *SD* = Standard Deviation

### Adjusted Associations between Emotion Regulation Difficulties and Smoking Status

Table 3 depicts the associations between ER difficulties (DERS total and subscale scores) and the adjusted odds of current smoking among adults with COPD, controlling for age, sex, depression, anxiety, and dyspnea severity. Consistent with prediction, DERS total scores were significantly associated with greater odds of current smoking (*p* <.001), after adjusting for all control variables. Each 10-point increase in DERS total scores (range 36 to 180) was associated with an approximately 20% increase in odds of current smoking. Of the DERS subscales, greater non-acceptance of emotions (*p* =.006), greater difficulties engaging in goal directed behaviors (*p* =.046), greater difficulties in emotional awareness (*p* =.014), greater difficulties accessing adaptive regulation strategies (*p* =.002), and greater difficulties in emotional clarity (*p* =.001) were associated with greater odds of current smoking.

**Table 3:** Results of adjusted logistic regression models examining the relationship between DERS total and subscales with smoking status (*n* = 309)

	AOR	SE	95% CI	*p*
Step 1: Controls				
Age	0.94	0.01	0.92, 0.96	<.001
Female sex	1.08	0.26	0.65, 1.79	.767
Depression	2.20	0.61	0.66, 7.32	.196
Anxiety	0.65	0.75	0.15, 2.80	.563
Dyspnea severity	1.16	0.13	0.89, 1.50	.271
Step 2: DERS				
Total	1.02	0.01	1.01, 1.04	.001
Non-acceptance	1.07	0.02	1.02, 1.12	.006
Goals	1.06	0.03	1.00, 1.13	.046
Impulse	1.05	0.03	0.99, 1.12	.096
Awareness	1.07	0.03	1.01, 1.13	.014
Strategies	1.08	0.03	1.03, 1.13	.002
Clarity	1.15	0.04	1.06, 1.24	.001

*Notes.* DERS = Difficulties in Emotion Regulation Scale, *AOR* = Adjusted Odds Ratio, *SE* = Standard Error, 95% CI = 95% Confidence interval. Separate logistic regression models were used to evaluate DERS total and subscale scores in relation to smoking status adjusted for age, sex, probable depression, probable anxiety, and dyspnea severity

### Adjusted Association between Emotion Regulation Difficulties and Daily Cigarette Use

Table 4 depicts the associations between ER difficulties (DERS total and subscale scores) and cigarettes smoked per day among adults with COPD, controlling for age, sex, depression, anxiety, and dyspnea severity. Contrary to our hypothesis, DERS total scores were not significantly associated with daily cigarette use (Δ*F* (1, 142) = 1.86, *p* =.175). Greater difficulties in awareness of emotions was associated with significantly more cigarettes smoked per day (Δ*F* (1, 142) = 5.00, *p* =.027), accounting for an additional 3.3% of the variance is cigarettes smoked per day. However, no other ER difficulties were significantly associated with cigarettes smoked per day in adjusted models (*p*s $\ge$ 0.05).

**Table 4:** Results of adjusted hierarchical linear regression models examining the relationship between DERS total and subscales with number of cigarettes smoked per day among smokers (*n* = 149)

	B	SE	95%CI	*p*
Step 1: Controls				
Age	0.10	0.06	-0.03, 0.22	.120
Female sex	-1.31	1.81	-4.88, 2.27	.472
Depression	-0.11	3.71	-7.44, 7.22	.976
Anxiety	0.33	4.50	-8.55, 9.22	.941
Dyspnea severity	-0.57	1.77	-4.07, 2.92	.746
Step 2: DERS				
Total	0.06	0.04	-0.03, 0.15	.175
Non-acceptance	-0.46	0.16	-0.36, 0.27	.775
Goals	0.16	0.23	-0.29, 0.62	.478
Impulse	0.12	0.20	-0.27, 0.51	.550
Awareness	0.42	0.18	0.05, 0.76	.027
Strategies	0.16	0.15	-0.14, 0.47	.290
Clarity	0.49	0.25	0.00, 0.98	.050

*Notes.* DERS = Difficulties in Emotion Regulation Scale, *SE* = Standard Error, 95% CI = 95% Confidence interval. Separate linear regression models were conducted to evaluate DERS total and subscale scores in relation to cigarettes smoked per day among smokers. All results are adjusted for age, sex, probable depression, probable anxiety, and dyspnea severity

## Discussion

The current study adds to the existing body of research on emotion dysregulation and smoking behaviors by examining difficulties in ER between smokers and non-smokers with COPD. Current smokers with COPD are particularly vulnerable to the negative health consequence of smoking and may represent a subgroup of cigarette-users who have been resistant to quitting smoking or who have been unsuccessful in their previous quit attempts. Consequently, these findings have the potential to facilitate smoking cessation efforts in COPD patients.

Consistent with hypotheses, smokers with COPD reported significantly greater overall ER difficulties compared to non-smokers with COPD. In relation to our exploratory aims, the results indicated significantly greater difficulties in each dimension of ER among smokers with COPD. In addition, the adjusted models revealed a robust association between overall ER difficulties and the odds of current smoking among adults with COPD. For example, in the current sample, individuals scoring one standard deviation above the mean on the DERS had approximately 47% greater odds of current smoking, even after controlling for age, sex, probable depression, probable anxiety, and dyspnea severity. This finding suggests that overall ER difficulties represents an important factor associated with smoking among adults with COPD, after accounting for well-established risk factors.

With the exception of the impulsivity dimension of ER, each facet of ER difficulties was associated with higher odds of smoking, even after controlling for age, sex, probable anxiety, probable depression, and dyspnea severity. These results are consistent with past research that suggests smoking is used as a method for regulating emotions [9, 21]. Additionally, the current study is one of few studies showing that specific dimensions of ER difficulties, including non-acceptance of emotions [21], lack of emotional clarity [18], difficulty engaging in goal-directed behaviors and limited access to ER strategies [37], contribute to smoking behaviors. Impulsivity may be less important in the maintenance of smoking behavior among adults who continue to smoke even after being diagnosed with a serious chronic respiratory disease. For example, the habitual nature of smoking across emotional experiences and the extent of nicotine dependence may play comparatively more important roles in the persistence of smoking in this population. The present findings inform ongoing research efforts designed to identify transdiagnostic processes in COPD [38] and specifically, provide the first indication that ER abilities might be implicated in smoking behaviors for those with COPD.

Contrary to our hypotheses, overall ER difficulties did not predict the number of cigarettes smoked per day among smokers with COPD. Among the specific domains of ER difficulties, only limited awareness of emotions predicted number of cigarettes smoked per day among smokers. These findings diverge from one study showing a direct association between negative affect regulation and number of cigarettes smoked [22]. However, our findings are consistent with prior substance use research. For example, while pre-use affect is associated with alcohol use in general it is not associated with the number of drinks consumed [39]. Additionally, a prior study found that only the impulse subscale of the DERS was associated with number of alcoholic drinks consumed, whereas ER deficits overall and subscales were associated with alcohol use more generally [40]. These might indicate that the *amount* of substance used is less dependent on ER. Past research has found that emotion dysregulation is predictive of cigarette dependency in a general [41] and substance use treatment seeking populations [37]. In consideration of these prior and current findings, our findings indicate that ER is generally associated with smoking dependency but not with daily smoking frequency.

Smoking remains one of the most significant health risks among individuals with COPD. Adults with COPD are at risk of detrimental functional outcomes [42] and comorbid mental health diagnoses [8]; with continuation of smoking being a particularly high-risk behavior among those with COPD, compounding negative outcomes. For example, individuals with COPD who smoke cigarettes have lower physical functioning, as well as greater risk of hospitalization and early mortality [5, 6, 43]. Despite these negative health consequences, many adults with COPD have difficulty quitting smoking. Indeed, this study and one previous study have reported high rates of current smoking (38 and 48%) among adults with COPD [4]. Prior studies have highlighted numerous barriers to smoking cessation, including beliefs about association between COPD and smoking [44], low motivation to change [45], and minimal hope of success [46]. Results of prior studies as well as the present findings suggest that ER may represent a viable treatment target for smoking cessation interventions among adults with COPD.

The present findings should be interpreted in the context of several important limitations. First, the present study used a cross-sectional design which precludes causal interpretation of the associations among study variables. For example, adults with COPD who have greater difficulties in ER may be more likely to smoke cigarettes to manage negative emotional experiences. Conversely, smoking may contribute to greater difficulties in ER. Second, although the present sample was recruited from a panel of individuals with chronic respiratory disease, the diagnosis of COPD was not confirmed with by a provider diagnosis or spirometry. Third, we assessed respondents’ current smoking status (smoking vs. not smoking) but did not assess smoking history. Consequently, we are not able to examine differences in ER difficulties among ever vs. never smokers. If evident, greater ER deficits among adults with a history of smoking compared to adults who have never smoked would further support the role ER deficits in smoking behavior. Last, cigarette consumption was assessed using a global self-report of daily cigarette smoking. Albeit a standard approach to assessment, future research should consider the inclusion of additional measures of smoking dependence, history of quit attempts, or intention to discontinue smoking to further elucidate the relation between emotion dysregulation and clinically relevant smoking indicators among current smokers with chronic lung conditions.

## Conclusions

Smoking is among the most important risk factors for medical complications and early mortality among adults with COPD [4]. Accordingly, smoking cessation is the primary target for medical and behavioral interventions among smokers with COPD. The present study provides preliminary data linking specific ER difficulties to smoking behavior among individuals with COPD. If corroborated by future research, these findings suggest that ER might be a potential target of interventions for smoking cessation among individuals with COPD. In light of the current findings, a critical first step would be increasing emotional awareness to help such individuals recognize the need for ER strategies, followed by equipping them with the abilities to access, select, and implement effective ER strategies. Specifically, prior research among smokers has indicated that targeting the use of adaptive ER strategies, and specifically, the use of cognitive reappraisal, resulted in reduction of cravings, increased willingness to experience smoking related thoughts, and longer periods before relapse [25]. ER treatment enhanced CBT has yielded high rates of abstinence and decreased daily cigarette smoking among individuals without chronic respiratory disease [47]. Future studies should examine the effectiveness of ER based interventions for current smokers with COPD.

## Electronic Supplementary Material

Below is the link to the electronic supplementary material.

Supplementary Material 1
